# Detection of *Candida* species in pregnant Chinese women with a molecular beacon method

**DOI:** 10.1099/jmm.0.000740

**Published:** 2018-04-20

**Authors:** Yanhong Zhai, Jing Liu, Li Zhou, Tongzhen Ji, Lingxin Meng, Yang Gao, Ran Liu, Xiao Wang, Lin Li, Binghuai Lu, Zheng Cao

**Affiliations:** ^1^​ Department of Laboratory Medicine, Beijing Obstetrics and Gynecology Hospital, Capital Medical University, Beijing, PR China; ^2^​ Department of Obstetrics, Beijing Obstetrics and Gynecology Hospital, Capital Medical University, Beijing, PR China; ^3^​ Triplex International Bioscience (China) Co., Ltd., Xiamen, Fujian, PR China; ^4^​ Central Laboratory, Beijing Obstetrics and Gynecology Hospital, Capital Medical University, Beijing, &nbsp;PR China; ^5^​ Laboratory of Clinical Microbiology and Infectious Diseases, Department of Pulmonary and Critical Care Medicine, China–Japan Friendship Hospital, Beijing, PR China

**Keywords:** vulvovaginal candidiasis, *Candida*, prevalence, pregnancy, susceptibility

## Abstract

**Purpose:**

*Candida* pathogens are commonly found in women and can cause vulvovaginal candidiasis (VVC), whose infection rate is further increased during pregnancy. We aimed to study the *Candida* prevalence and strain distribution in pregnant Chinese women with a molecular beacon assay.

**Methodology:**

From March 2016 to February 2017, a total of 993 pregnant women attending routine antenatal visits at the Beijing Obstetrics and Gynecology Hospital were enrolled. For *Candida* detection and identification, a unique molecular beacon assay was presented and compared with a traditional phenotypic method. Antifungal susceptibility was tested with the following agents: 5-flucytosine, amphotericin B, fluconazole, itraconazole and voriconazole.

**Results:**

The prevalence of *Candida* was found to be 21.8 % when using the molecular method and 15.0 % when using the phenotypic method. The distribution of the *Candida* spp. was listed in order of decreasing prevalence: *Candida albicans* (79.8 %), *Candida glabrata* (13.5 %), *Candida parapsilosis* (3.7 %), *Candida krusei* (2.2 %) and *Candida tropicalis* (1.1 %). We found that 90.7 % of the *Candida* detection results were consistent between the molecular and the phenotypic methods. In the cases where the sequencing analyses for the *Candida* isolates resulted in inconsistent identification, the molecular method showed higher sensitivity than the phenotypic method (96.0 vs 64.6 %). *C. albicans*, *C. glabrata* and *C. parapsilosis* were essentially susceptible to all five antifungal agents tested, whereas *C. tropicalis* and *C. krusei* were susceptible to voriconazole and amphotericin B.

**Conclusion:**

By exhibiting good sensitivity and specificity, the molecular assay may offer a fast and accurate *Candida* screening platform for pregnant women.

## Introduction

Vulvovaginal candidiasis (VVC) is the second most common cause of vaginitis, affecting 70–75 % of women of reproductive age at least once during their lifetime [1]. About 5–8 % of women suffer recurrent VVC (RVVC), experiencing four or more infection episodes per year [2]. According to a recent study involving Chinese RVVC participants, the duration of the patients’ complaints varied from 6 months to 10 years (mean duration: 22.3 months) [3]. More importantly, previous studies showed that *Candida* species could be isolated from at least 20 % of asymptomatic healthy women, while the infection rate increased to 30 % during pregnancy [4, 5].

The majority of VVC infections result from *Candida albicans*, accounting for 85–95 % of all cases. Among the remaining non-*albicans* species, *Candida glabrata* is the second most common species. *C. glabrata*, together with other relatively uncommon species, such as *Candida parapsilosis*, *Candida tropicalis* and *Candida krusei*, also causes VVC, with fewer clinical symptoms and more resistance to treatment [1, 5]. Interestingly, *C. glabrata* infections are more frequently associated with RVVC [1].

The primary complaints of VVC-infected patients often include itching, burning, redness, swelling and cottage-cheese-like vaginal discharge. However, these clinical symptoms are non-specific to VVC and their severity varies depending on demographic and behavioural factors during pregnancy, which is a common risk factor for VVC infection [6]. It is found that a higher prevalence of symptomatic VVC infection and RVVC, and a less effective therapeutic response, were seen in pregnant compared to non-pregnant women [7]. It is hypothesized that the estrogen-rich environment during pregnancy increases the glycogen content in vaginal tissue and enhances the adherence of *Candida* spp. to vaginal epithelial cells, resulting in more frequent VVC infection and resistance to treatment [5]. Complications associated with VVC during pregnancy can affect both the pre- and postpartum stages. There are several reports in which intra-amniotic infection caused by *C. albicans* and *C. glabrata* resulted in preterm membrane ruptures or preterm labour [8]. Congenital candidiasis of the newborns can be acquired *in utero* or during delivery. Even in asymptomatic pregnant women, recurrent *Candida* colonization has been shown to be associated with increased preterm delivery and low birth weight, when compared to women with normal or intermediate flora [9]. These impaired pregnancy outcomes are further aggravated if *Candida* colonization occurs in the second trimester [10]. In a randomized trial of clotrimazole, it was proven that treating asymptomatic women with *Candida* colonization can prevent preterm birth.

The detection of *Candida* spp. relies on traditional phenotypic tests such as micromorphology, chromogenic medium (CHROM Agar Candida), germ tube tests, microculture in agar cornmeal and automated biochemical confirmation (VITEK 2) [11–13]. These phenotypic tests usually take more than 48 h to complete identification, with more or less subjective judgment, leading to lower diagnosis accuracy and potentially delayed treatment. However, there is no reliable serological or antigen detection technique for VVC diagnosis. Here we report a molecular beacon method for rapid VVC detection and identification. This method is based on the PCR technique and relies on the hybridization and opening of a beacon-shaped probe to its complementary target nucleic acid sequence [14]. In the present study, the molecular beacon method for *Candida* spp. identification was evaluated in 993 pregnant women at 16 gestational weeks or older. Strain distribution and antifungal susceptibility testing in *Candida* spp., including *C. albicans*, *C. glabrata*, *C. parapsilosis*, *C. tropicalis* and *C. krusei*, were assayed in the recruited patients.

## Methods

### Patients and sample collection

From March 2016 to February 2017, a total of 993 pregnant women (16–40 weeks of gestation) attending routine antenatal visits at the Beijing Obstetrics and Gynecology Hospital were recruited to our study. The clinical symptoms in the reproductive tract of recruited patients were not questioned because of privacy concerns. The subjects were excluded if their condition met one of the following exclusion criteria: topical medication in genital tubes within a week, diagnosis of VVC, or diagnosis of gestational diabetes mellitus or any kind of immune deficiency. The research protocol was approved by the Institutional Research Review Board of the Beijing Obstetrics and Gynecology Hospital. All participants enrolled in the study signed consent forms.

Two samples of cervical/vaginal mucosa were collected from each patient with sterile cotton-tipped swabs. One swab was used for phenotypic tests and the other swab was used for DNA preparation in the molecular beacon assay.

### Molecular beacon assay

DNA was extracted from each sample using the DNA-Free-RY kit (Triplex International Bioscience, People’s Republic of China) according to the manufacturer’s instructions. Extracted DNA was stored at −20 °C until use, with the concentration being measured with NanoDrop (Thermo Scientific) at 280/260 nm. Based on the distinct sequences of the internal transcribed spacer 2 (ITS2) of *Candida* spp., the primers and hybridization probes were designed using Primer Premier 5.0 software (PREMIER Biosoft) (Table S1, available in the online version of this article). The molecular beacon assay was carried out with the Applied Biosystems 7500 Real-Time PCR (Thermo Fisher). To identify each of the five *Candida* spp., the species-specific PCR reaction mixtures (40 µl) were set up as follows: 5 ng template DNA, 0.2 mM dNTPs, 0.6 µM primers mix, 0.1 µM probe, 2.5 U *Taq* polymerase (TaKaRa Bio, Inc) and 0.2 U uracil-N-glycosylase (TaKaRa Bio, Inc). Forty amplification cycles were performed (20 s at 94 °C and 45 s at 55 °C) for each reaction tube. The analytical sensitivity of the molecular beacon assay was estimated by serially diluting template genomic DNA prepared from standard cultures of *Candida*. To confirm the specificity of the primers and probes in differentiating the above five *Candida* spp., standard strains of *C. albicans*, *C. glabrata*, *C. parapsilosis*, *C. tropicalis* and *C. krusei*, together with another 19 fungi or bacteria species (*Streptococcus bovis*, *Streptococcus agalactiae*, *Staphylococcus epidermidis*, *Staphylococcus aureus*, *Aspergillus fumigatus*, *Aspergillus flavus*, *Aspergillus niger*, *Enterococcus faecalis*, *Pseudomonas aeruginosa*, *Neisseria gonorrhoeae*, *Treponema pallidum*, *Chlamydia trachomatis*, *Ureaplasma urealyticum*, *Escherichia coli*, *Candida sake*, *Pichia guilliermondii*, *Candida colliculosa*, *Candida intermedia* and *Candida kefyr*) that are common pathogens found in women’s reproductive tracts, were purchased from ATCC or the China General Microbiological Culture Collection Center (CGMCCC) (Table S2). For each standard strain, the genomic DNA was extracted from their cultures, reaching 10^6^ colony-forming units (c.f.u.) ml^−1^, and this was followed by the molecular beacon assay described previously.

### Phenotypic identification and antifungal susceptibility test

For the phenotypic identification method, each vaginal/cervical swab was streaked on a chocolate agar plate supplemented with vancomycin for 24–48 h at 35 °C. The suspicious yeast isolates were further inoculated to CHROMagar *Candida* Media plates (Jingzhang Technology, Tianjin, People’s Republic of China), with incubation at 35 °C for another 24 h. The unresolved isolates from CHROMagar plates were alternatively identified by the Vitek 2 compact system (bioMérieux, France). The antifungal *in vitro* susceptibility test was performed with the ATB FUNGUS 3 (bioMérieux, France) strip in a semi-solid medium under similar conditions to those in the reference method [15]. According to the manufacturer’s instructions, the minimum inhibitory concentrations (MICs) for amphotericin B (AMB), fluconazole (FCA), itraconazole (ITR) and voriconazole (VRC) were determined by visually interpreted growth scores (0–4). For AMB, the MIC corresponds to the lowest concentration enabling complete growth inhibition (score 0), whereas for FCA, ITR and VRC it corresponds to the lowest concentration of the antifungal agent with which a score of ‘0’, ‘1’, or ‘2’ is obtained. For 5-flucytosine (5FC), antifungal susceptibility is interpreted as ‘susceptible’, ‘susceptible dose-dependent’, or ‘resistant’, based upon the growth scores obtained at the two concentrations of 5FC.

Wherever necessary, the remaining DNA extracted from the vaginal swabs or colonies in the phenotypic assay was sent out for sequencing in the nuclear ribosomal internal transcribed spacer (ITS) region, which is considered to be a universal DNA barcode marker for fungi [16]. The ITS sequencing service was provided by Sangon Biotech Co., Ltd (Shanghai, People’s Republic of China) and this employed the Sanger sequencing technique (amplification primers in sequencing: 5′-TCCGTAGGTGAACCTGCGG-3′, and 5′-TCCTCCGCTTATTGATATGC-3′).

### Statistical analyses

A chi-square test was carried out to compare the prevalence difference for *Candida* spp. when determined by molecular and phenotypic methods. The data analyses were performed with IBM SPSS 21.0, with *P*<0.05 considered to be statistically significant.

## Results

### Prevalence of *Candida* colonization and strain distribution

In the present study, the primers and probes in the molecular beacon method were designed specifically to detect and identify *C. albicans*, *C. glabrata*, *C. parapsilosis*, *C. tropicalis* and *C. krusei*, based on the ITS region of each organism. Each of the five *Candida* species-specific PCR methods exhibited an analytical sensitivity of 50 copies ml^−1^ or lower. None of them exhibited cross-reaction with each other or any of the other 19 organisms tested (Table S2), showing the high specificity of the method as a whole.

Of the 993 pregnant women screened for *Candida* pathogens at our hospital, 21.8 % (216/993) tested positive by the molecular method and only 15.0 % (149/993) tested positive by the phenotypic method. The distribution of the five *Candida* spp. is listed in order of decreasing prevalence (with the average prevalence from the two methods included in parentheses): *C. albicans* (79.8 %), *C. glabrata* (13.5 %), *C. parapsilosis* (3.7 %), *C. krusei* (2.2 %) and *C. tropicalis* (1.1 %) (Table 1). The prevalence of the five *Candida* spp. did not differ statistically between the molecular and the phenotypic methods, according to the chi-square test results (Table 1).

**Table 1.** Prevalence and strain distribution of different *Candida* spp. in 993 patients

**Species**	**Method (prevalence)**				***P (*χ^2^)**
	**Molecular**		**Phenotypic**		
	**No. of cases**	**%**	**No. of cases**	**%**	
*C. albicans*	172	79.6	119	79.9	0.96
*C. glabrata*	32	14.8	18	12.1	0.46
*C. parapsilosis*	4	1.9	8	5.4	0.06
*C. tropicalis*	3	1.4	1	0.7	0.52
*C. krusei*	5	2.3	3	2.0	0.85
Total	216	100.0	149	100.0	

The statistical analyses were performed with the chi-square test, with *P*<0.05 indicating statistical significance.

### Comparison of methods

The methodological comparison in the present *Candida* spp. screening study is summarized in Table 2. The identification results for the molecular and the phenotypic methods agreed by as much as 90.8 % (901/993). As expected, the majority (85.0 %, 766/901) of the consistent results tested negative for *Candida*. For the consistent results, the prevalence and distribution of the each *Candida* species were similar to those of the total population (Table 1), with *C. albicans* (83.0 %, 112/135) and *C. glabrata* (15.5 %, 21/135) being the two leading species. With DNA being either aliquoted from the molecular assay or freshly extracted from *Candida* colonies, ribosomal ITS sequencing was employed as the ‘gold standard’ method to confirm both consistent and inconsistent identification results. Of the subgroup with consistent identification, all four *Candida* spp., including *C. albicans*, *C. glabrata*, *C. parapsilosi*s and *C. tropicalis*, were positively confirmed by sequencing with randomly selected *Candida* isolates (Table 2). Because only limited template DNA was left over from the molecular assay, it was not possible to confirm all of the inconsistent results by ITS sequencing.

**Table 2.** Comparison of methods for *Candida* spp. identification

**Count**	**Methods**		
	**Molecular**	**Phenotypic**	**Sequencing (*n*=53)**
Consistent results (*n*=901)			
112	*C. albicans*	*C. albicans*	*C. albicans* (*n*=3)
21	*C. glabrata*	*C. glabrata*	*C. glabrata* (*n*=5)
1	*C. parapsilosis*	*C. parapsilosis*	*C. parapsilosis* (*n*=1)
1	*C. tropicalis*	*C. tropicalis*	*C. tropicalis* (*n*=1)
0	*C. krusei*	*C. krusei*	nd
766	Negative	Negative	nd
Inconsistent results (*n*=92)			
60	*C. albicans*	Negative	*C. albicans* (*n*=21)
			*Cladosporium* sp. (*n*=2)
9	*C. glabrata*	Negative	*C. glabrata* (*n*=5)
3	*C. parapsilosis*	Negative	nd
1	*C. tropicalis*	Negative	nd
5	*C. krusei*	Negative	*C. krusei* (*n*=3)
5	Negative	*C. albicans*	*C. albicans* (*n*=4)
1	Negative	*C. glabrata*	*Saccharomyces cerevisiae* (*n*=1)
3	Negative	*C. parapsilosis*	*C. parapsilosis* (*n*=2)
			*C. lusitaniae* (*n*=1)
0	Negative	*C. tropicalis*	nd
2	Negative	*C. krusei*	*C. krusei* (*n*=1)
1	*C. glabrata*	*C. parapsilosis*	*C. glabrata* (*n*=1)
1	*C. glabrata*	*C. krusei*	*C. glabrata* (*n*=1)
1	*C. tropicalis*	*C. albicans*	*C. tropicalis* (*n*=1)
993	Total		

nd, Not determined.

Seventy-eight samples tested positive for *Candida* by the molecular method but negative by the phenotypic method. Thirty-one of the 78 samples were sequenced and essentially all returned results in that agreed with those determined by the molecular method, except for the two that were interpreted as being *C. albicans*. The two isolates erroneously identified as *C. albicans* were actually contamination incidences from *Cladosporium* sp., which has previously been reported as an unusual contamination during cervical smear examinations [17].

Out of the 11 samples that were positively identified by the traditional phenotypic method but negatively identified by the molecular method, two identification mistakes were made according to the corresponding sequencing results. One was actually *Saccharomyces cerevisiae*, but was interpreted as *C. glabrata*, while the other was *C. lusitaniae*, but was mistakenly confused with *C. parapsilosis*. Only three isolates had discrepant positive results between the molecular and the phenotypic methods. Later sequencing results proved that the molecular identification was correct for all of them (Table 2). Considering that not all the inconsistent results were confirmed by sequencing, the apparent sensitivity for the molecular and the phenotypic methods was, respectively, 96.0 % (214/223) and 64.6 % (144/223), whilst the specificity was 99.7 % (768/770) for both methods (Table 3).

**Table 3.** Clinical performance of the two *Candida* identification methods

	**Molecular**			**Phenotypic**		
	***n***	**Sensitivity**	**Specificity**	***n***	**Sensitivity**	**Specificity**
TP	214	96.0 %	99.7 %	144	64.6 %	99.7 %
FN	9			79		
TN	768			768		
FP	2			2		
Total	993			993		

TP, true positive; FN, false negative; TN, true negative; FP, false positive.

Sensitivity (%) was calculated as 100* TP/(TP+FN).

Specificity (%) was calculated as 100*TN/(TN+FP).

### Antifungal susceptibility test

Antifungal susceptibility testing was carried out on the 139 *Candida* isolates from the phenotypic assay. In the antifungal susceptibility test, *C. albicans*, *C. glabrata* and *C. parapsilosis* were essentially susceptible to all the five antifungal agents tested, whereas *C. tropicalis* (*n*=1) and *C. krusei* (*n*=1) were susceptible to VRC and AMB (Table 4).

**Table 4.** Antifungal susceptibilities of *Candida* spp. from colonized patients

**Antifungal agents**		***Candida* spp.**				
		***C. albicans* (*n*=116)**	***C. glabrata* (*n*=18)**	***C. parapsilosis* (*n*=3)**	***C. tropicalis* (*n*=1)**	***C. krusei* (*n*=1)**
5-Flucytosine						
*n* (%)	S	112 (96.6)	18 (100.0)	3 (100.0)	1 (100.0)	–
	SDD	–	–	–	–	–
	R	4 (3.4)	–	–	–	1 (100.0)
Amphotericin B						
*n* (%)	S	115 (99.1)	18 (100.0)	3 (100.0)	1 (100.0)	1 (100.0)
	SDD	–	–	–	–	–
	R	1 (0.9)	–	–	–	–
Fluconazole						
*n* (%)	S	105 (90.6)	16 (88.8)	3 (100.0)	–	–
	SDD	4 (3.4)	1 (5.6)	–	1 (100.0)	–
	R	7 (6.0)	1 (5.6)	–	–	1 (100.0)
Itraconazole						
*n* (%)	S	99 (85.3)	7 (38.9)	2 (66.7)	–	–
	SDD	8 (6.9)	5 (27.8)	–	–	1 (100.0)
	R	9 (7.8)	6 (33.3)	1 (33.3)	1 (100.0)	–
Voriconazole						
*n* (%)	S	112 (96.6)	18 (100.0)	3 (100.0)	1 (100.0)	1 (100.0)
	SDD	1 (0.9)	–	–	–	–
	R	3 (2.5)	–	–	–	–

The antifungal susceptibility test was carried out on the *Candida* isolates from phenotypic assays. Only the *Candida* isolates that yielded consistent *Candida* spp. identification results or had been confirmed by sequencing for strain identity were included.

S, susceptible; SDD, susceptible dose-dependent; R, resistant.

## Discussion

The mainstream technique for *Candida* identification still relies on the traditional phenotypic methods, which can take 2–4 days to yield results. In this study, a molecular beacon method was used to detect and identify the presence of *Candida* using vaginal swabs over a shorter period of time (less than 48 h). Our molecular identification system contains five independent PCR reactions designed for *C. albicans*, *C. glabrata*, *C. parapsilosis*, *C. tropicalis* and *C. krusei*. Relying on species-specific probes that accurately recognize the ITS2 sequence, our assay was highly specific and no cross-reactivity was observed between the *Candida* spp or with any of the other 19 commonly observed fungal or bacterial pathogens. A similar strategy has been employed successfully in the rapid molecular assay for *Candida dubliniensis* identification, as reported by Park *et al.* in 2000 [18].

This study revealed a prevalence of *Candida* colonization of 21.8 % in pregnant Chinese women by the molecular method and one of 15.0 % by the phenotypic method. With both symptomatic and asymptomatic participants being recruited, the overall *Candida* isolation rate reported in the present study was comparable to that observed in pregnancy by Sangare *et al*. (22.7 %) [19], in which both patients with and without clinical signs were included.

As expected, *C. albicans* and *C. glabrata* were the two most frequently observed *Candida* spp. during pregnancy. Their combined prevalence was 93–95 %, similar to that (96 %) revealed by a retrospective study in which 3141 non-pregnant Chinese women were included [12]. *C. albicans* is the most common *Candida* species isolated in VVC patients, with a prevalence ranging within 65–97 % in women. Recently, a high genetic heterogeneity of *C. albicans* in pregnancy was observed between individuals [20]. This finding may be explained by microevolution and could be used to predict novel phenotypes such as antifungal resistance and improved virulence. As the second most common pathogen in VVC, *C. glabrata* was more pathogenic in immune-compromised patients than in healthy hosts [21]. The combined prevalence of the other three *Candida* spp., including *C. parapsilosis*, *C. tropicalis* and *C. krusei*, was found to be 5.6 % when using the molecular method and 8.1 % when using the phenotypic method, with these values being slightly higher than those in previously published data (2.6 %) from a 10-year study concerning non-pregnant Chinese women [12]. This prevalence difference may be explained by the physiological changes during pregnancy that are described above. Interestingly, Sangare *et al*., from Burkina Faso, reported that the combined prevalence of *C. tropicalis* and *C. krusei* was as high as 26.9 % for local pregnant women, showing the huge influence of geographical and climatic factors.

As shown in Table 2, 90.7 % of the *Candida* detection results were consistent between the molecular and the phenotypic methods. The majority of the inconsistent results (84.8 %, 78/92) belonged to the ‘molecular positive/phenotypic negative’ results pattern. With limited DNA being left over from the molecular typing assay, 31 ‘molecular positive/phenotypic negative’ isolates were sent for sequencing, with 29 being confirmed positively. Surprisingly, the two isolates identified as *C. albicans* turned out to be *Cladosporium* sp. by sequencing. Unfortunately, we found that the amplification primers and probe designed for *C. albicans* had 73–85 % sequence similarity in the genome of *Cladosporium* sp. according to the blast search results (https://blast.ncbi.nlm.nih.gov/Blast.cgi). *Cladosporium* sp. is an anemophilous fungus and is considered to be an exogenous fungal body in humans. *Cladosporium* sp. has been found to contaminate conventional cervical smears, posing a challenge to cytopathologists [17]. Common sources of this fungus are house plants kept in examination rooms or damp areas such as basements and water pipes. There were 11 ‘molecular negative/phenotypic positive’ isolates, nine of which were sequenced. According to the sequencing results, one *S. cerevisiae* isolate was misinterpreted as *C. glabrata* and one *C. lusitaniae* isolate was misinterpreted as *C. parapsilosis*, showing the limitations of the phenotypic method for uncommon and non-*Candida* species. Indeed, although they are not commonly observed, both *S. cerevisiae* and *C. lusitaniae* have been reported in a VVC study [12]. The three ‘double positive’ cases with inconsistent identification results were sent for sequencing, which later confirmed that the results obtained the molecular method were all correct. The misidentification in those three cases may have been introduced by human errors, especially when the morphological and colour appearances were similar for some *Candida* species.

It is relatively well documented that *C. albicans* is susceptible to most azoles, and that non-*albicans* species are less sensitive to azoles [5, 22]. A similar observation was made in our susceptibility study. However, due to the heterogeneity of the *in vitro* susceptibility techniques, various observations have been made in these kinds of studies. For example, in the antifungal study by Brandolt *et al.* [11], *C. albicans* displayed >50 % resistance to both FCA and ITR [11]. Another confounding factor that might have contributed to the unexpected susceptibility testing results is the misuse of over-the-counter azole drugs. An earlier study showed that the actual diagnosis rate for VVC in self-diagnosed women was only 33.7 % [23], which further led to excessive exposure to azoles and subsequent drug resistance [24]. Similar to the observation previously reported [25], VRC and AMB were among the most effective antifungal agents against *C. krusei*, which was resistant to FCA in our susceptibility test (Table 4).

In summary, a highly sensitive and specific molecular method was introduced to detect and identify five *Candida* species. Compared to the traditional phenotypic method, the molecular method was more sensitive and involved a shorter turnaround time in the clinical laboratory. Lastly, for the first time, the prevalence and strain distribution of *Candida* spp. in pregnant Chinese women was reported, along with the *in vitro* antifungal susceptibility tests. *C. albicans* and *C. glabrata* were the two most common species in the present study and were essentially sensitive to all the antifungal drugs tested. However, due to the lack of confirmatory VVC diagnosis in the current study, no conclusion about *Candida* prevalence and its association with asymptomatic colonization and symptomatic VVC patients could be drawn at this point. Therefore, the ability of the present *Candida* molecular beacon identification assay to differentiate true infection from simple colonization still needs to be evaluated in future studies.

## Supplementary Data

120 Supplementary File 1
