# Supplementary material for: Detection of Candida species in pregnant Chinese women with a molecular beacon method
Source: J Med Microbiol. 2018 Apr 20;67(6):783–9. doi: 10.1099/jmm.0.000740 (PMC6096925; doi:10.1099/jmm.0.000740)
Supplement: Supplementary File 1 [file jmm-67-783-s001.pdf]

Table S1. Sequences of the primers and probes for the molecular beacon assays

Candida spp.	**Forward primer**	**Reverse primer**	**Probe**
C. albicans	GGCGCTCAAGCCT GCTACT	ACGCCTGCCTACT CGTGAA	ATACTTCACCGTGATTG CTGTTTTGACGC
C. glabrata	CGAGAACAACTCC GATTCTATAACC	TGGACTCAGAATC AGCATTTTCA	CCAATGCGACCAACGAA AAACACATG
C. parapsilosis	CATTTGCTCAATT GTATGCTGATTT	CGCAGCATCACGT TTCCA	CCCCAATCAATTTTGTTT TCCCACACTTG
C. tropicalis	ATAACAGGTCTGT GATGCCCTTAGA	CGCTGGCTCCGTC AGTGTA	TTCTGGGCCGCACGCGC
C. krusei	CGGCCGGGTCTTT CCTT	CCTGCTTTGAACA CTCTAATTTCCT	CCTCGGGCGAACCAGGA CGATTAC

All the primer and probe sequences are displayed displayed in the 5´to 3´direction.

Table S2. Specificity in the molecular beacon assays for *Candida* identification

Species	Reference number		Ct values for real-time PCR				
	CGMCCC	ATCC	*C. albicans*	*C. glabrata*	*C. parapsilosis*	*C. tropicalis*	*C. krusei*
C. albicans	2.4159		19	ND	ND	ND	ND
C. glabrata	2.3983		ND	18	ND	ND	ND
C. parapsilosis	2.3989		ND	ND	18.5	ND	ND
C. tropicalis	2.3967		ND	ND	ND	17	ND
C. krusei	2.1857		ND	ND	ND	ND	19
Streptococcus bovis	1.1624		ND	ND	ND	ND	ND
Streptococcus agalactiae		13813	ND	ND	ND	ND	ND
Staphylococcus epidermidis	1.1757		ND	ND	ND	ND	ND
Staphylococcus aureus	1.2465		ND	ND	ND	ND	ND
Aspergillus fumigatus	3.6005		ND	ND	ND	ND	ND
Aspergillus flavus	3.6151		ND	ND	ND	ND	ND
Aspergillus niger	3.6478		ND	ND	ND	ND	ND
Enterococcus faecalis	1.2024		ND	ND	ND	ND	ND
Pseudomonas aeruginosa	1.2387		ND	ND	ND	ND	ND
Neisseria gonorrhoeae		19424	ND	ND	ND	ND	ND
Treponema pallidum		BAA-2642SD	ND	ND	ND	ND	ND
Chlamydia trachomatis		VR-571B	ND	ND	ND	ND	ND
Ureaplasma urealyticum		27618	ND	ND	ND	ND	ND
Escherichia coli	1.1772		ND	ND	ND	ND	ND
Pichia guilliermondii	2.3943		ND	ND	ND	ND	ND
Candida sake	2.3955		ND	ND	ND	ND	ND
Candida colliculosa	2.2003		ND	ND	ND	ND	ND
Candida intermedia	2.2603		ND	ND	ND	ND	ND
Candida kefyr	2.2295		ND	ND	ND	ND	ND

The specificity assays were carried out with the genomic DNA from the above standard microorganisms.

The PCR reaction setup was described in the Methods section.

Ct: threshold cycle in real-time PCR; ND: not detectable within the 40 repeating PCR cycles

CGMCCC: China General Microbiological Culture Collection Center
